# Possible Dangers of the Bubble Fountain

**Published:** 1917-01

**Authors:** 


### POSSIBLE DANGERS OF THE BUBBLE FOUNTAIN.

The propaganda in the interest of public health has succeeded in introducing numerous innovations into our daily life. Most of these have improved sanitary conditions; some have helped to eradicate disease and to promote hygienic efficiency. The dangers to health which lurk in the common use of articles that find an application in the everyday life of man with respect to his person have become more clearly recognized with the development of bacteriology. Dentists' and barbers' utensils, the common towel, and the public drinking cup are believed to harbor a menace because of the possibility of transmitting infection from person to person through the successive use of the same device without special purification or sterilization for each application.

Laws have been framed to prohibit the use of the common drinking cup and are being widely enforced in this country. As a result, the "individual" or single service cup, usually made of paper, has come into use; and where suitable water supply is available, the bubble fountain has been generally installed. In the latter device it has seemed as if the application of sanitary science had almost reached hygienic perfection; for what could be more obvious than the freedom of the ever changing bubbling stream of pure water from contamination through the evanescent contact with the mouth?

The circumstance of an epidemic of streptococcus tonsillitis two years ago in one of the dormitories of the University of Wisconsin unexpectedly directed suspicion to the bubble fountains in the building. The water pressure in them was so low that it was scarcely possible to drink from the bubbler without touching the metal portions with the lips. An examination of the fountains showed them to be heavily contaminated with streptococci. Positive results were obtained from the surface of the fountain, from the inside and from the water discharged, but the city water supply by which they were operated gave no evidence of these organisms. These facts led to an extensive bacteriologic investigation of the hygiene of the bubble fountain in general by Pettibone, Bogart and Clark, of the University of Wisconsin Laboratory of Medical Bacteriology. From this it appears that the bubble fountains may become a factor in transmitting disease.

The facts of the Wisconsin investigation are surprising as well as unexpected. A survey of all fountains of the university showed the presence of streptococci in over fifty per cent. of the total number. These bacteria varied in abundance from a few chains to an almost pure culture obtained by swabbings from fountains in the epidemic-ridden dormitory. In an experimental bubble fountain, *Bacillus prodigiosus* when introduced either by means of a pipet or by the moistened lips remained in the water from two to 135 minutes, depending partly on the height of the "bubble."

The explanation of this finding seems to be clear. Most of the organisms are flushed away in the water stream; but some remain dancing in the column much as a ball dances on the garden fountain, even though the bubble be increased to the impracticable height of four inches. To avoid this difficulty, always present in the vertical column of spouting water, a simple fountain with a tube at an angle of fifty degrees from the vertical was constructed. *B. prodigiosus* was never found in the culture plates from this type of fountain, even when samples were taken immediately after the intentional introduction of the organism.

Danger disguised in the cloak of safety is a menace of the most potent sort, particularly when it receives the approbation of health authorities in the way that the bubble fountain has shared it. The Wisconsin investigators believe that a jet of water from a tube erected at an angle of fifteen degrees or more from the vertical and with an adequate collar guard to prevent possible contact with the orifice is adequate to prevent micro-organism from "dancing'' on the column of water. The construction of a safe bubble fountain is thus made practicable, and the problem of furnishing water uncontaminated by human lips even to a constant succession of thirsty persons is apparently solved anew.—*Journal A. M. A.*, November 11, 1916.

